# From Game to Concert: Exploratory Listening in ‘Stardew Valley: Festival of Seasons’ Concert Tour

**DOI:** 10.3390/bs15050667

**Published:** 2025-05-13

**Authors:** Natalie P. Miller, Elizabeth Hellmuth Margulis

**Affiliations:** Department of Music, Princeton University, Princeton, NJ 08544, USA; margulis@princeton.edu

**Keywords:** music, exploratory listening, multimedia, immersion, presence, musical imaginings, live concerts, video games

## Abstract

Exploratory listening encompasses the various ways, contexts, and levels of attention with which listeners engage with their sonic environment. This paper presents findings from qualitative research conducted with audience members during the *Stardew Valley: Festival of Seasons* concert tour. During these events, attendees encountered music from the widely successful indie video game, reorchestrated in a new context. Just as the game encourages exploration through open-ended gameplay, the concerts prompt listeners to explore how the rearranged music refers to and diverges from its use in the game. Findings suggest that attendees deployed their attention to divergent aspects of the music. While some attendees focused on specific musical aspects like recognizable melodies and instrumentation, others focused on the broader audiovisual and community aspects of the performance. Results also indicate that highly immersed listeners experience diverse thoughts, including those not directly about the immediate musical content. Positioning music-evoked imaginings as a way listeners become immersed in musical experiences, we report on how exploratory listening shapes the dynamics of attention, immersion, and enjoyment within musical and audiovisual contexts.

## 1. Introduction

Defined as ‘the psychological experience of being engrossed in the media world’ (Tukachinsky, 2014, p. 4), psychological immersion describes states of engaged attention, in both live and technologically mediated contexts. Foundational work by Witmer and Singer (1998) conceptualize this state of engagement as ‘a psychological state characterized by perceiving oneself to be enveloped by, included in, and interaction with an environment that provides a continuous stream of stimuli and experiences’ (p. 227). In media environments, states of immersion involve temporal and spatial dissociation with the real world (Curran, 2018; Haywood & Cairns, 2006; Jennett et al., 2008).

Music plays a significant role in shaping immersion in mediated contexts like film (Lehman, 2013; Cohen, 2015), television (Tan et al., 2013), music videos (Vernallis, 2013; Korsgaard, 2017), and other multimedia formats (Cook, 2004). Recent work in multimedia concert research (Hunt, 2024; Greenfield-Casas, 2023) explores how multimedia concerts gamify live performance, reshaping audience engagement. Taking music from media to the concert hall, ‘cine-concerts, or live-to-picture events, feature a live performance of an underscore (and on occasion source music) accompanying a screening of a motion’ (Okazaki, 2020, p. 3). Similarly, live concerts of video games often ‘feature large screens upon which game footage is projected to accompany the music’ (Hunt, 2024, p. 649). These concerts rearrange music from their initial media context for orchestral or chamber groups (Greenfield-Casas, 2023) and often embed gamified elements that include audience members as performers (Hunt, 2024). Blurring the boundaries between live performance and digital media, these concerts guide listeners’ musical attention in novel ways (Okazaki, 2020; Sekar, 2022), requiring them to negotiate familiar music in new performance environments.

Live concerts encourage audiences to employ exploratory listening behaviors as they ‘manage their attentional focus and levels of processing in search of meaningful carriers of meaning’ (Reybrouck et al., 2024, p. 7). Drawing on predictive frameworks, listeners use exploratory listening behaviors to become immersed in the concert experience (Pianzola et al., 2021). Through highly attentive modes of listening (Weining et al., 2025; Weining, 2022; Wald-Fuhrmann et al., 2021), audience members bring the prior audiovisual contexts, memories, and emotions they have come to associate with the music to the immersive experience of live performance. In these ways, live concerts promote deep engagement with music, encouraging attendees to become highly immersed in the experience.

This paper reports on a survey study of attendees at several *Stardew Valley: Festival of Seasons* concerts in 2024. Best known for being ‘a humble, intimate farming adventure about the monotony of domestic life,’ (White, 2018), the farming simulation video game *Stardew Valley* (ConcernedApe, 2016) transports players into the game world in an immersive role-playing experience. Given the game’s flexible structure, the cognitive load involved in the gameplay is likely lower than many other game genres (e.g., puzzle, strategy, and action games). Reflecting a growing trend for live performances to feature music from multimedia franchises (Greenfield-Casas, 2023; Hunt, 2024), the *Festival of Seasons* concert series adapted music from *Stardew Valley* (ConcernedApe, 2016) for the concert hall by arranging the electronic soundtrack for a 12-piece chamber ensemble. Marketed as an ‘intimate, immersive live concert featuring fresh arrangements of the most cherished songs from the game’s mesmerizing soundtrack’, (SOHO Live, 2024) the tour sold out 63 concerts across 13 countries worldwide (Brandt, 2024).

Survey responses indicate that listeners became highly immersed in the experience, engaging in exploratory listening behavior to make sense of *Stardew Valley*’s soundtrack in its new concert context. These results are indicative of the way multimedia concerts encourage exploratory listening behaviors, underscoring the significant impact that distinct levels of attention and modes of listening shape listening experiences in live concert settings (Weining et al., 2025; Swarbrick et al., 2024; Kreuzer et al., 2025). Extending Reybrouck et al. (2024), this paper examines how exploratory listening behaviors shape listeners’ musical interpretations and broader patterns of thought.

## 2. Materials and Methods

### 2.1. Participants

All data collection procedures in the study were assessed and approved by the Princeton Institutional Review Board. Participants were 163 concertgoers (mean age = 28.7, SD of 7.6; 92 women, 52 men, 18 nonbinary and multiple gender identities, 1 prefer not to respond) who responded to surveys distributed at five performances of *Stardew Valley: Festival of Seasons* in Atlanta (n = 57), Austin (n = 91) and Philadelphia (n = 15) in 2024. Of the 163 respondents, 85 reported some level of disagreement with the statement ‘I am an experienced musician’, while 78 reported some level of agreement (1–6 scale).

### 2.2. Materials

The questionnaire was hosted on Qualtrics and distributed at the concerts via flyers that featured a QR code link to the full survey. All participation was voluntary and no compensation was provided. After completing an IRB-approved consent form that confirmed they were over 18 years of age, respondents completed the four sections of the survey. The first assessed their degree of immersion with 11 questions excerpted from the engagement subscale of the Sense of Presence Inventory (Lessiter et al., 2001). The second section asked listeners to assess if the concert performance felt more similar or different from the initial in-game music (1–6 scale). Listeners were then asked to describe the perceived similarities or differences. The third section asked listeners to report on the type of thoughts they sustained while listening, by checking as many boxes as they wanted from a list of 10 of the thought types shown in Table 1, adapted slightly from Jakubowski et al. (2024). If they checked the box for thoughts about the music, memories, or fictional scenes, they were additional asked to provide a free text description of what they experienced. The full survey is available in Supplementary Materials.

## 3. Results

### 3.1. Musical Familiarity

**Figure 1:**
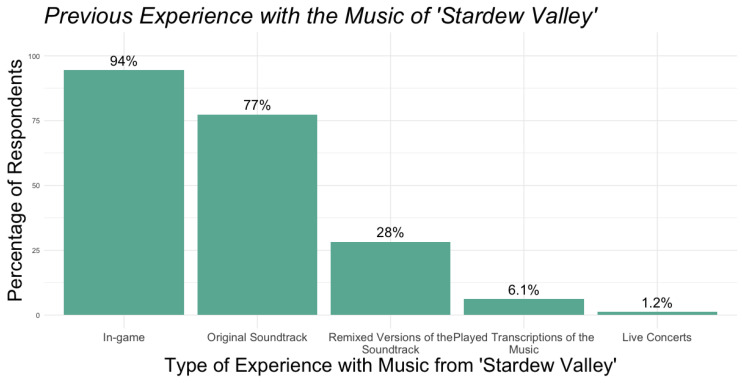
Respondents’ engagement with the music of *Stardew Valley* prior to the concerts.

Overall, most respondents indicated high levels of familiarity with the music of *Stardew Valley* prior to attending *Festival of Seasons* concerts, with 94% of participants indicating that they had been at least somewhat familiar with the music before attending. To better assess how listeners developed this high level of familiarity with the music, we asked respondents to select all the different ways they engaged with the music prior to the concert. Figure 1 summarizes the distributions of these engagements.

### 3.2. Immersion

Overall, respondents indicated very high rates of enjoyment, engagement, and immersion as gauged through the ITC-Sense of Presence Inventory (Lessiter et al., 2001). The mean immersion score was 4.3 (SD = 0.5, IQR = [4.1, 5]) on a five-point scale. Throughout the survey, attendees frequently commented on how concert elements affected their degree of immersion in the experience. Table 2 highlights representative examples.

### 3.3. Comparing Musical Interpretations

Even though most respondents were familiar with the music prior to the concert, they differed substantially when asked to rate how similar the concert music felt to their prior experiences with the music of *Stardew Valley*, on a scale from 1 (completely different) to 6 (completely the same). Almost half of the respondents, 43%, felt that the music was more different than similar and the remaining 57% felt that the music felt more similar than different. These responses highlight the broad range of similarity judgments, despite participants’ overwhelming familiarity with the music.

Directly following the Likert similarity judgment, participants were asked to describe the perceived musical similarities or differences. Respondents who rated the music as being more different than similar (1–3 on the scale) were asked to describe differences. Respondents who rated the music as being more similar than different (4–6 on the scale) were asked to describe similarities. To better understand what musical features listeners focused on, we used natural language processing to analyze trends in their text descriptions. Responses were grouped into two categories: difference descriptions and similarity descriptions. We did not omit any complete responses that discussed both similarities and differences as manual inspection revealed that less than 10% of the responses described both similarities and differences. All responses were lemmatized and stop words were removed including ‘which’, ‘this’, ‘I’m’, and ‘their’, as well as task-related words like ‘different’, ‘similar’, ‘familiar’, and ‘stardew’.

**Figure 2:**
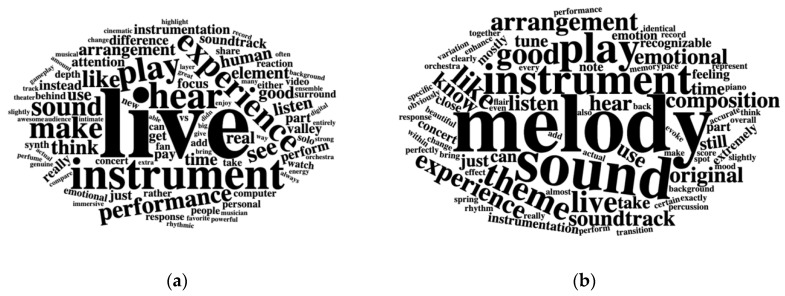
(**a**) Word cloud for descriptions of musical differences across in-game and concert experience. (**b**) Word cloud for descriptions of musical similarities between the contexts. In both word clouds, all words were lemmatized, stop words were removed, and only words with more than one occurrence are visualized.

The most common words for both groups are displayed in Figure 2. The group of respondents who highlighted similarities employed many music-specific words like ‘melody’, ‘instrument’, ‘theme’, ‘sound’, and ‘soundtrack’. They seemed to focus on ‘recognizable’ and specific musical content that was shared between the two contexts. In comparison, descriptions of musical differences emphasized the ‘live’ and experiential nature on the concert, focusing on the performers and setting rather than on the specific musical material.

After this visual inspection of word clouds, we used the tidytext package in R (Silge & Robinson, 2016) to calculate term frequency–inverse document frequency (TF-IDF) values for each word in the corpus of responses. Adding nuance to the frequency counts used to create Figure 2 (the above word clouds), this technique identifies the most prominent terms used for both groups of responses, allowing us to assess which musical features listeners prioritized when making their comparisons. We treated each group (different and similar) as entire documents when generating the TF-IDF values for each lemmatized word. Rather than focusing on individual responses, we focused on the group level to uncover broader patterns in word usage across these two groups.

**Figure 3:**
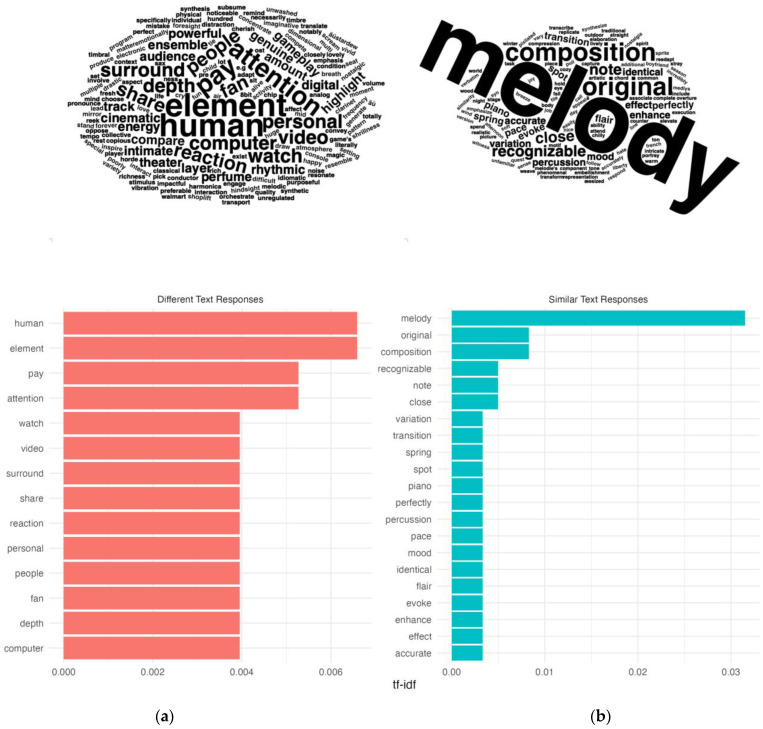
(**a**) Word cloud for all words in descriptions of differences with non-zero TF-IDF values, with corresponding bar chart for words with highest TF-IDF values. (**b**) Word cloud for all words in descriptions of similarities with non-zero TF-IDF values, with corresponding bar chart for words with highest TF-IDF values.

Through this technique, we found striking differences in word usage between the two response groups. Figure 3 summarizes the words with the highest TF-IDF values for each category. People who felt the music was markedly different from the in-game context more often used words like ‘human’, ‘attention’, ‘watch’, ‘video’, and ‘people’ to explain the perceived differences. The prominence of these words may suggest that listeners’ musical attention was often pulled to audiovisual and community aspects of the performance. However, people who felt the music was more similar to the in-game music more frequently emphasized the ‘melodies’, ‘notes’, ‘emotions’, and ‘recognizable’ aspects of the music. While it may not be surprising that listeners prioritized melodic information in their similarity assessments, these findings may suggest that listeners’ attention may have been more directly focused on specific musical material, musical motives, and emotional associations with the music. Table 3 and Table 4 consider the broader context of the terms with the largest value TF-IDF values, highlighting representative quotes that contain top terms for differences and similarities.

### 3.4. Patterns of Thought

**Figure 4:**
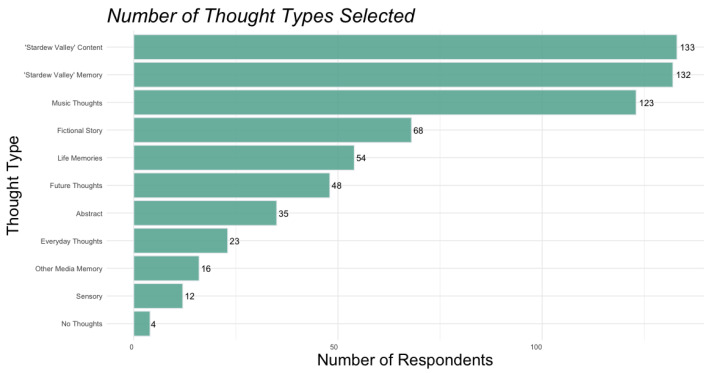
Distribution of thought types selected by respondents.

Trends in thought types experienced by audience members resemble larger trends of music-evoked imaginings (Jakubowski et al., 2024). Participants at this live concert event experienced a wide range of thought types with a substantial increase in thoughts about future or personal plans (29.4% of respondents) when compared to Jakubowski et al. (2024), which differed from the present study in modality (headphones), familiarity (unfamiliar music), and genre (classical, electronic, popular). The distribution of reported thoughts is summarized in Figure 4, shown in order from most prevalent to least prevalent type of thought. Together with the high level of reported immersion, this broad spread of thought types shows a clear comingling of high immersion with diversity of thought types. Notably, our data show a high volume of thoughts that were not directly about the music, including fictional stories, autobiographical memories, and thoughts about the future.

### 3.5. Relation of Immersion and Number of Thought Types

**Figure 5:**
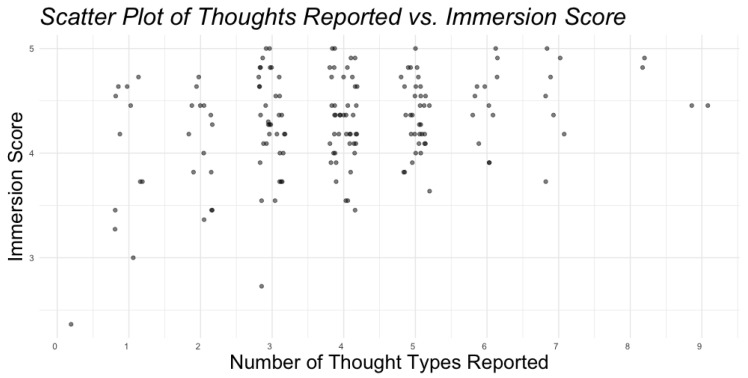
Relationship between the number of thought types reported and immersion score, with each dot representing a single respondent.

We conducted a Spearman rank-order correlation to assess the relationship between the number of thought types reported and immersion score, as shown in Figure 5. There was a significant positive correlation between the two variables (ρ = 0.22, *p* = 0.004), indicating that participants who reported a greater variety of thoughts tended to report a higher degree of immersion.

### 3.6. Correlation Between Different Thought Types

**Figure 6:**
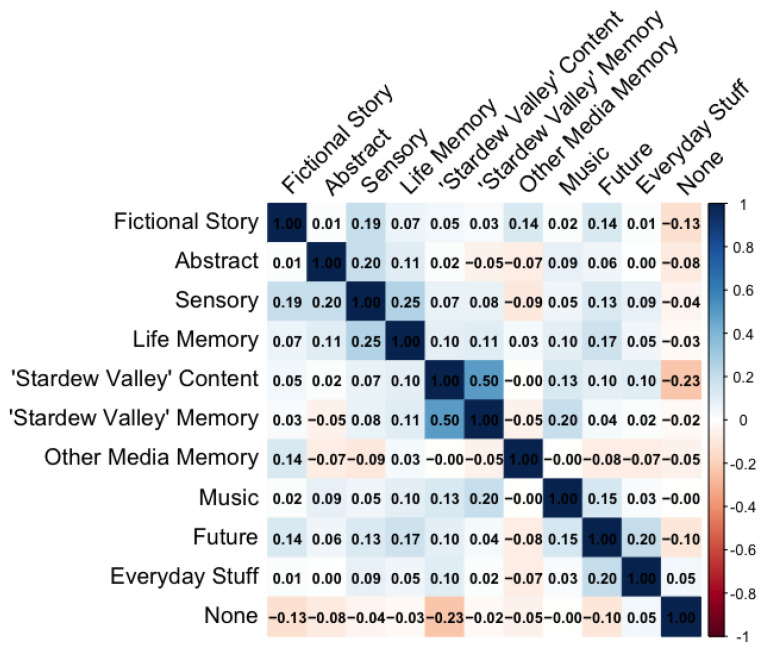
Correlation matrix of co-occurring thought types during the concert.

Figure 6 summarizes the degree to which individual thought types co-occurred for concertgoers, calculated by performing Pearson’s correlations on pairs of binary variables. All significant correlations are summarized in Table 5. The strongest correlation (φ = 0.5, *p* < 0.001) occurred between life memories associated with *Stardew Valley* and specific thoughts about content from the game, indicating that participants who experienced one of these thought types also often experienced the other. Recalling life memories was correlated with future thoughts (φ = 0.17, *p* = 0.03), potentially suggesting a clustering of autobiographical thoughts cued by the game’s music. The positive correlation between thoughts about the music and *Stardew Valley* memories (φ = 0.2, *p* = 0.01) supports this interpretation.

### 3.7. Relation of Immersion and Specific Thought Types

**Figure 7:**
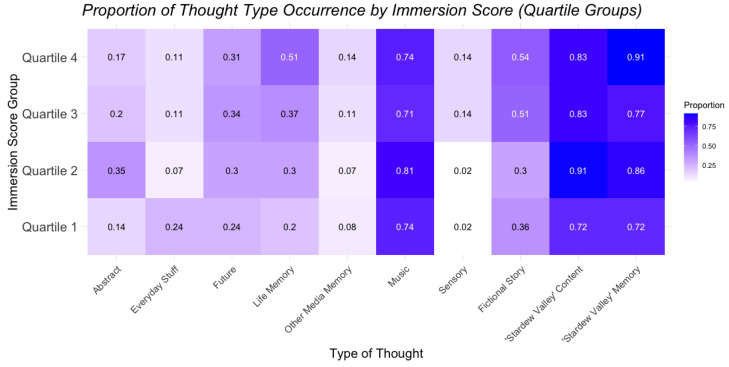
Proportion of thought type occurrence across immersion quartile groups.

Figure 7 explores the relationship between the presence of different thought types and the listener’s degree of immersion (split into four levels based on quartiles). It suggests that concertgoers experiencing an increased degree of immersion were also experiencing more life memories and fictional imaginings. Levels of musical training and fandom engagement did not coincide significantly with patterns of thought types.

Throughout their responses, participants reported a high degree of immersion, varied musical descriptions, and diverse patterns of thoughts. Although many were highly familiar with the music prior to the concert, listeners’ musical attention varied widely. Some respondents focused on the live and community aspect of the performance, while others allocated their attention toward specific musical features. Thought patterns were similarly varied, with a high prevalence of autobiographical memories and imagined fictional narratives. Notably, the degree of immersion correlated with the number of thought types. These findings also suggest that the nature of thought experiences may be shaped by the co-occurrence of multiple thought types and by individual differences in immersion.

## 4. Discussion

These findings prompt further critical examination of how immersion and engagement are defined, especially for musical experiences. While these concepts are often described as states of focused attention, our results indicate that highly immersed listeners actually experience diverse thoughts, including those not directly about the immediate musical content. Responses show that the highest immersion coincided with the greatest number of thought types, especially fictional imaginings and autobiographical memories. These findings suggest that immersive experiences may not necessarily lead to a singularly focused engagement with music but might prompt listeners instead to become immersed in the thoughtscape that is triggered by the music.

This necessitates a shift in understanding how music facilitates immersion. Rather than only promoting engaged attention on the sound itself, music can cue absorbing internal imaginings like fictional stories, autobiographical memories, and memories of related media. Previous research has shown that music-evoked imaginings are closely tied to sound (Margulis et al., 2022a, 2022b; McAuley et al., 2021), shaped by cultural contexts (Margulis et al., 2022b) and prevalent in many genres of music (Jakubowski et al., 2024; van der Walle et al., in press). Extending this body of research, our findings suggest that these diverse patterns of thought occur in live concert contexts for listeners highly familiar with the musical material, and they coincide with high degrees of immersion in the concert experience. These findings align with multidimensional approaches to musical absorption, emphasizing how absorbed states relate to other experiential dimensions such as concurrent visual imagery (Vroegh, 2024) and mind wandering (Swarbrick et al., 2024).

These results indicate that imaginings cued by live concert contexts with relatively lengthy musical pieces may differ from imaginings studied in most behavioral research, in contexts such as listening to recordings of brief musical excerpts over headphones. For example, 48 (29.4%) of respondents at the *Festival of Seasons* concerts reported that they thought about future plans during the concert, while the recorded cues in van der Walle et al. (in press) rarely generated any reports of thoughts about the future. Moreover, our data showed almost exclusively positive correlations between different thought types while correlations between thought types in van der Walle et al. (in press) were much more varied, including significant negative correlations between fictional imaginings and music-related thoughts and fictional imaginings and autobiographical memories. These differences may suggest that specific listening contexts may engender different degrees of immersion by prompting different patterns of music-evoked imaginings.

A key implication from this study is that immersed states may not depend on attending exclusively to the music itself, but can also include attending closely to an imagining or memory cued by the music. These findings support a fluid and multidimensional approach to immersion and engagement, creating space to consider how dynamic changes in attention shape the listening experience. In this view, immersion might involve dynamic shifts between attentional modes—alternating between present-moment engagement with the sound and internally generated imagery or memory. Frameworks like Pianzola et al.’s (2021) nested approach to levels of presence and immersion and Omigie and Mencke’s (2024) time-varying model of musical engagement may be especially helpful in examining the fluidity of immersive states and their relation to music-evoked imaginings. Future work should explore the relationship between imagined scenes and musical immersion more directly.

This study’s limitations point to the importance of future research. The use of a self-selected participant sample introduces the potential for sampling bias, which may have influenced the frequency and types of thoughts reported. As such, the results may not fully generalize to broader or more diverse concert audiences. Additionally, the concert series focused exclusively on music from a single video game soundtrack. While this offered a consistent musical framework, it constrains the generalizability of the findings. Future research should investigate a wider variety of multimedia concerts—spanning media formats, franchises, performance structures, and audience demographics—to better understand how musical familiarity, visual context, and gameplay complexity interact to shape immersion and musical thought. Expanding the scope in this way will help clarify how diverse multimedia contexts structure audience engagement and musical attention.

Together, these findings underscore how listeners engaged in ‘open-minded and active process’ (Reybrouck et al., 2024, p. 1) as they explored the concerts’ sonic landscape. By experiencing familiar music in a novel concert environment, concertgoers drew on highly attentive modes of listening (Wald-Fuhrmann et al., 2021; Weining et al., 2025). Referencing their prior experiences with the music, they drew on predictive frameworks to make sense of the remediated musical content. Deploying their attention to different attributes of the experience, some attendees focused more on the live, human quality of the performances while other listeners focused on specific musical material and melodies. Our findings indicate significant overlap between the processes of exploratory listening, immersion, and thought patterns, suggesting that each play a crucial role in shaping listeners’ aesthetic judgements and emotional-affective outcomes. As one respondent summarizes, ‘I can hear people’s lives getting better after every song’.

## Figures and Tables

**Table 1:** Response options for thoughts experienced during the concert, adapted from Jakubowski et al. (2024).

Thought Type Selection Options
I imagined a fictional story or scene.
I imagined abstract shapes, colours, and/or patterns.
I imagined smells, tastes, and/or other sensory sensations.
I recalled memories of experiences from my life.
I recalled specific scenes, images, or gameplay from “Stardew Valley”.
I recalled memories from playing “Stardew Valley”.
I had thoughts about the music.
I had thoughts about the future or personal plans.
I was thinking about everyday stuff.
Other (please describe below):

**Table 2:** Representative free-text descriptions from respondents, describing their degree of immersion.

Quotes From Respondents Describing Immersion
The arrangements brought depth that took me outside of the seat in the theater. I was transported and felt extremely happy!
The music took me right to the outdoors in cool breezes of spring, warm days of summer, cozy fall afternoons, and chilly winter nights, perfectly represented by a little pixilated world.
It felt more beautiful and intimate. The music was more in-depth and I felt like I was really there.
The concert elevated the music from background soft music to a full out of body experience. The orchestra completely transformed the experience into an immersive emotional experience.
It felt more powerful in-person with live music, and it was the only thing I was paying attention to instead of having it in the background
While playing the game, I felt the music mainly become a background element as I focused more on what I was doing in game. While sitting in the theater, specifically for the music, I felt much more engaged in the music.
I paid more attention to the details of the arrangement and how they adapted it to live performance
it felt more cinematic! when I’m playing the game, I’m not paying attention so much to the music either

**Table 3:** Representative difference responses using words with the five largest TF-IDF values.

Selected Difference Responses Containing Largest TF-IDF Terms
The music in a concert setting felt more like a collective experience (e.g., audience interactions, reactions, etc.) than when I listen to the music on my own. It was fun seeing my personal reactions and feelings mirrored among hundreds of people. It was awesome! It added an extra layer of love for the music of Stardew Valley that I’ll cherish forever.
More personal, more imaginative, more experienced.
The fact that it was being perfumed live and that everyone else in the room shared the same experience with this game
The live human element of it all
I paid more attention to the details of the arrangement and how they adapted it to live performance
Getting to hear live instruments is very different from listening to synthesizers on the computer or from a game console
The richness of the orchestral instruments! And the human-ness! So good. It brought the score alive.
Volume, frequency and vibrations from the analog instruments produces a more physical reaction than the emotional response of the digital video game soundtrack.
The atmosphere and the sound was more genuine rather than computer generated
It felt more intimate and personal when I could see the music being performed live
It was better, there’s a certain amount of feeling you get to experience watching them perform some of your favorite songs
The emotional response to the music was stronger when hearing it during the concert as compared to playing the game. The music feels more powerful in a live, multi-instrument performance with an audience surrounding me.
While playing the game, I felt the music mainly become a background element as I focused more on what I was doing in game. While sitting in the theater, specifically for the music, I felt much more engaged in the music.
The energy behind the live performance. It was great to watch the themes being shared across multiple players and to experience the new instrumentation.
There are clear timbral and rhythmic qualities that exist in a video game like‚ Stardew Valley Taking that experience and adding that extra human element of performance can often highlight those differences. Most notably was the use of either differing timbres (horn & violin, namely) to convey a solo that in game most closely resembles a harmonica. Another example is through some of the difficult rhythmic and melodic patterns that—due to it being a pre-programed performance in game—we get used to hearing in their perfect conditions. Those specific parts are often not idiomatic for the instruments chosen to represent them, leading to some noticeable mistakes mid-performance. However, I didn’t feel that either of these necessarily took away from the experience. Instead, I always enjoy seeing just how you are able to expand upon the musical experience that so many have through video games using these live ensemble settings.
It was very cool to watch people perform the music, adding a human element. Also interesting to listen without the distraction of the game

**Table 4:** Representative similarity responses using words with the five largest TF-IDF values.

Selected Similarity Responses Containing Largest TF-IDF Terms
The rhythm and melody
The songs were recognizable and still held the same emotional value, but the orchestra has a different sound than the traditional soundtrack.
The notes and the composition were about as identical as you could reasonably get with a live performance instead of synthesizing.
it was easily recognizable
The songs and compositions were familiar to me
Live versions of most songs were very close to recorded originals but obviously will sound slightly different.
The music itself was very close to the original composition. However, it felt more emotional live.
The music was very recognizable
Use of similar instruments and very accurate to the original melodies
The arrangements didn’t stray much at all from the original score. Including the instruments used. They were almost perfectly accurate.
The melodies and notes were all mostly the same.
The common melodies were the same, but some parts were different due to the nature of the original score being transcribed to instruments
The composition, obviously, but I’d have been disappointed if I couldn’t recognize the songs
The emotional appeal that the compositions are able to evoke
When you’d focus on the background or close your eyes you could feel similarities with the time in game.

**Table 5:** Significant Pearson’s correlations between co-occurring thought types.

Thought Type 1	Thought Type 2	Phi	*p* Value
Sensory	Fictional Story	0.19	0.02
Sensory	Abstract	0.2	0.01
Life Memory	Sensory	0.25	0.001
Future	Life Memory	0.17	0.03
‘Stardew Valley’ Memory	‘Stardew Valley’ Content	0.5	<0.001
None	‘Stardew Valley’ Content	−0.23	0.003
Music	‘Stardew Valley’ Memory	0.2	0.01
Everyday Stuff	Future	0.2	0.01

## Data Availability

The raw data supporting the conclusions of this article will be made available by the authors on request.

## Supplementary Materials

The following supporting information can be downloaded at: https://www.mdpi.com/article/10.3390/bs15050667/s1, The following supporting information includes the set of survey questions reported on in this paper.
