# Supplementary material for: From Game to Concert: Exploratory Listening in ‘Stardew Valley: Festival of Seasons’ Concert Tour
Source: Behav Sci (Basel). 2025 May 13;15(5):667. doi: 10.3390/bs15050667 (PMC12109176; doi:10.3390/bs15050667)
Supplement: Supplementary file 1 [file behavsci-15-00667-s001.zip › behavsci-3532623-supplementary.pdf]

From Game to Concert: Exploratory Listening in ‘Stardew Valley: Festival of Seasons’ Concert Tour

Natalie Miller[1]* and Elizabeth H. Margulis[2]

Start of Block: Consent

Q1 Thank you for participating in our research study!

In this survey, you will answer questions about your experience with the music of *Stardew Valley: Festival of Seasons*. The survey should only take approximately 5 minutes to complete.

Before you begin, please make sure: That you are at least 18 years of age. That you are completing this survey either **during intermission** or **within 24 hours of the concert's completion**.

---

*

Q4 Please indicate your age in years.

____

End of Block: Consent

Start of Block: ITC-SoPI

Q5 Please select the performance(s) you have attended or are currently attending.

- ☐ Philadelphia, 5pm, June 1st, 2024 (1)
- ☐ Philadelphia, 8pm, June 1st, 2024 (2)
- ☐ Austin, 3pm, June 15th, 2024 (5)
- ☐ Austin, 7pm, June 15th 2024 (6)
- ☐ Atlanta, 7pm, July 14th 2024 (7)
- ☐ Other: (4) ____

Q66 Please indicate when you are completing this survey in relation to the concert.

- ○ During intermission (1)
- ○ Within 1 hour of the concert's conclusion (2)
- ○ Between 1 hour and 24 hours after the concert's conclusion (3)
- ○ More than 24 hours after the concert's conclusion (please elaborate below): (4) ____

Page Break

Q7 Please indicate **HOW MUCH YOU AGREE OR DISAGREE** with each of the following statements by selecting just ONE of the numbers using the 5-point scale below.

AFTER my experience of the concert...

	1 - Strongly disagree (1)	2 - Disagree (2)	3 - Neither agree nor disagree (3)	4 - Agree (4)	5 - Strongly Agree (5)
Q1 from ITC-SoPI (1)	○	○	○	○	○
Q2 from ITC-SoPI (2)	○	○	○	○	○
Q3 from ITC-SoPI (3)	○	○	○	○	○
Q4 from ITC-SoPI (4)	○	○	○	○	○

Page Break

Q8 Please indicate **HOW MUCH YOU AGREE OR DISAGREE** with each of the following statements by selecting just ONE of the numbers using the 5-point scale below.

DURING my experience of the concert...

	1 - Strongly disagree (1)	2 - Disagree (2)	3 - Neither agree nor disagree (3)	4 - Agree (4)	5 - Strongly Agree (5)
Q5 from ITC-SoPI	○	○	○	○	○
Q6 from ITC-SoPI	○	○	○	○	○
Q7 from ITC-SoPI	○	○	○	○	○
Q8 from ITC-SoPI	○	○	○	○	○
Q9 from ITC-SoPI	○	○	○	○	○
Q10 from ITC-SoPI	○	○	○	○	○
Q11 from ITC-SoPI	○	○	○	○	○

End of Block: ITC-SoPI

Start of Block: Attention

Q9 How familiar are you with the music of *Stardew Valley*?

- 1 -- Extremely unfamiliar (1)
- 2 -- Mostly unfamiliar (2)
- 3 -- Somewhat more unfamiliar than familiar (3)
- 4 -- Somewhat more familiar than unfamiliar (4)
- 5 -- Mostly familiar (5)
- 6 -- Extremely familiar (6)

Page Break

Q10 How did the concert performance of this music feel when compared to your prior experiences listening to the music of *Stardew Valley*?

- ◯ 1 -- Completely different (1)
- ◯ 2 -- Mostly different (2)
- ◯ 3 -- Somewhat more different than similar (3)
- ◯ 4 -- Somewhat more similar than different (4)
- ◯ 5 -- Mostly the same (5)
- ◯ 6 -- Completely the same (6)

Page Break

Display This Question:

If similardifferentlikert = 4 -- Somewhat more similar than different

Or similardifferentlikert = 5 -- Mostly the same

Or similardifferentlikert = 6 -- Completely the same

Q11 Please elaborate on this response. What about the music felt **the most similar**?

____

Display This Question:

If similardifferentlikert = 1 -- Completely different

Or similardifferentlikert = 2 -- Mostly different

Or similardifferentlikert = 3 -- Somewhat more different than similar

Q12 Please elaborate on this response. What about the music felt **the most different**?

____

Page Break

Q13 Which of the following did you imagine or think of while listening to the music? Please select all that apply.

- ☐ I imagined a fictional story or scene. (10)
- ☐ I imagined abstract shapes, colours, and/or patterns. (22)
- ☐ I imagined smells, tastes, and/or other sensory sensations. (23)
- ☐ I recalled memories of experiences from my life. (24)
- ☐ I recalled specific scenes, images, or gameplay from "Stardew Valley". (30)
- ☐ I recalled memories from playing "Stardew Valley". (21)
- ☐ I recalled memories from media such as films, TV, or video games other than "Stardew Valley". (25)
- ☐ I had thoughts about the music. (26)
- ☐ I had thoughts about the future or personal plans. (27)
- ☐ I was thinking about everyday stuff. (28)
- ☐ No thoughts. (29)
- ☐ Other (please describe below): (19) ____

Page Break

Display This Question:

If thoughtsselection = I imagined a fictional story or scene.

Or thoughtsselection = I imagined abstract shapes, colours, and/or patterns.

Or thoughtsselection = I imagined smells, tastes, and/or other sensory sensations.

Or thoughtsselection = I recalled memories of experiences from my life.

Or thoughtsselection = I recalled specific scenes, images, or gameplay from "Stardew Valley".

Or thoughtsselection = I recalled memories from playing "Stardew Valley".

Or thoughtsselection = I recalled memories from media such as films, TV, or video games other than "Stardew Valley".

Or thoughtsselection = I had thoughts about the future or personal plans.

Or thoughtsselection = I was thinking about everyday stuff.

Or thoughtsselection = Other (please describe below):

Or Or thoughtsselection Text Response Is Not Empty

Q14 Please describe the general thoughts, memories or imaginings you had during the concert.

____

Display This Question:

If thoughtsselection = I had thoughts about the music.

Q15 Please describe the general thoughts, memories or imaginings you had during the concert **specifically related to the music.**

____

Display This Question:

If thoughtsselection = I had thoughts about the music.

Q25 Please describe the general thoughts, memories or imaginings you had during the concert **about the music of "Dance of the Moonlight Jellies,"** if applicable.

____

End of Block: Attention

Start of Block: Demographics

Q16 Which of the following options best describes your gender identity?

- ▢ Man (1)
- ▢ Nonbinary (2)
- ▢ Woman (3)
- ▢ I prefer not to answer (4)
- ▢ I prefer to self describe: (5) ____

Page Break

Q17 Please select how much do you agree or disagree with the following statements.

	1 -- Strongly disagree (1)	2 -- Disagree (2)	3 -- Somewhat disagree (3)	4 -- Somewhat agree (4)	5 -- Agree (5)	6 -- Completely agree (6)
I am an experienced player of "Stardew Valley." (1)	o	o	o	o	o	o
I feel connected to the larger "Stardew Valley" community. (4)	o	o	o	o	o	o
I feel comfortable as a part of the "Stardew Valley" community. (5)	o	o	o	o	o	o
I frequently engage with online content about "Stardew Valley". (7)	o	o	o	o	o	o
I avidly participate in online or in-person "Stardew Valley" fandom and community. (6)	o	o	o	o	o	o
I am an experienced musician. (8)	o	o	o	o	o	o

Page Break

Q18 Before the concert, how did you engage with the music of *Stardew Valley*? Please select all that apply.

- ▢ Attended prior live concerts of this music (1)
- ▢ In-game while playing "Stardew Valley" (4)
- ▢ Listened to the original soundtrack (5)
- ▢ Listened to remixed versions of the soundtrack (6)
- ▢ Played transcriptions or versions of the soundtrack on my own instrument (7)
- ▢ Other: (8) ____

Page Break

Q19 Is there anything else you would like to share about this survey or your experience with the music of Stardew Valley during, prior to, or after the concert?

____

End of Block: Demographics
